# A Novel Adaptive Cuckoo Search for Optimal Query Plan Generation

**DOI:** 10.1155/2014/727658

**Published:** 2014-08-14

**Authors:** Ramalingam Gomathi, Dhandapani Sharmila

**Affiliations:** ^1^Department of Computer Science and Engineering, Bannari Amman Institute of Technology, Sathyamangalam 638401, India; ^2^Department of Electronics and Instrumentation Engineering, Bannari Amman Institute of Technology, Sathyamangalam 638401, India

## Abstract

The emergence of multiple web pages day by day leads to the development of the semantic web technology. A World Wide Web Consortium (W3C) standard for storing semantic web data is the resource description framework (RDF). To enhance the efficiency in the execution time for querying large RDF graphs, the evolving metaheuristic algorithms become an alternate to the traditional query optimization methods. This paper focuses on the problem of query optimization of semantic web data. An efficient algorithm called adaptive Cuckoo search (ACS) for querying and generating optimal query plan for large RDF graphs is designed in this research. Experiments were conducted on different datasets with varying number of predicates. The experimental results have exposed that the proposed approach has provided significant results in terms of query execution time. The extent to which the algorithm is efficient is tested and the results are documented.

## 1. Introduction

Today, most of the data we require is available to us in the form of web pages. The web pages contain documents which are linked with each other. Humans can easily read these web pages, but the machine has difficulty in understanding the meaning of the web pages. This leads to the emergence of the semantic web. Many data models exist for storing the semantic web data. One of the fundamental data models is the RDF. It is a language for representing semantic web data. Each statement in RDF is represented using a set of triples which contains subject, predicate, and object. Three types of elements can be defined in RDF: resources represented by URI and blank nodes and literals represented by data values. A subject in a triple can be either a resource or a blank node. An object can be any element but predicate can be only a resource. For example, consider three infinite sets *R*, *B*, and *D* called resources, blank nodes, and literals. An RDF triple (subject, predicate, and object) is an element of (*R* ∪ *B*) × *R* × (*R* ∪ *B* ∪ *D*). An RDF graph is a set of RDF triples.

To query RDF, several query languages exist. The most popular of them is the SPARQL protocol and RDF query language (SPARQL). A SPARQL can query RDF datasets from many data sources. It can match both basic and complex graph patterns from multiple sources and can also filter the results using filter expressions. Different types of queries exist in SPARQL. But we use only SELECT queries with one or more WHERE clauses in the research. An encouraging research area in this perspective is the determination of optimal query paths. A query path refers to the order in which different parts of a query is executed. The query execution time is directly related with the query path. An efficient algorithm for finding an optimal query path can thus contribute to quick and efficient querying mechanism.

In the semantic web's perspective, some research has been done already in this area. Algorithms like [1] Iterative improvement (II) algorithm, simulated annealing (SA) also called two-phase optimization (2PO) algorithm, and genetic algorithm (GA) were used to address the problem of query path optimization. Traditional query optimization algorithms exist but as an alternative; there exist the soft computing techniques for optimizing the queries.

A metaheuristic algorithm is an iterative generation process which combines different concepts for exploring the search space and finds near-optimal solutions. These algorithms are approximate and usually nondeterministic. Metaheuristic algorithms are best suitable for NP-hard optimization problems. They give better quality solutions than heuristic methods. Query optimization is an NP-hard problem. As an alternate to the traditional optimization methods, we can optimize the query by using metaheuristic algorithms which are the best choice for solving NP-hard problems.

The paper is organized as follows.  Section 2 describes the literature review of work done in the related area.  Section 3 explains the original Cuckoo search algorithm.  Section 4 defines the solution space, encoding methodology, and the fitness function for the problem of query optimization.  Section 5 outlines the implementation of Adaptive Cuckoo search algorithm to determine the optimal query paths.  Section 6 summarizes the datasets and the experimental results.  Section 7 sketches the conclusion.

## 2. Related Work

The fundamental concepts related to efficient processing [2] of SPARQL query language were studied in the literature. A total complexity analysis for all operator fragments of the SPARQL query language was considered. Methods were designed for semantic optimization of SPARQL queries. The main idea behind semantic query optimization was, given a query and a set of integrity constraints, to find minimal (or more efficient) queries that are equivalent to the original query on each database instance that satisfies the constraints.

Semantic web data management needs efficient querying of large scale RDF triples. To determine the optimal query plan, a main-tree-shaped algorithm [3] was proposed in the literature which collects a set of RDF statistics for estimating the cost of the query plan. The first step of the algorithm converts every node into the execution subtree and the second step enumerates all the execution trees by treating each node as the root node and then choosing the tree with the minimum execution cost as the optimal execution plan. Experimental results have proved that the algorithm performs well for querying large scale RDF triples in terms of execution time.

A semantic query optimization approach [4] was designed to optimize query plans of heterogeneous multidatabase systems. The approach provides global as well as local optimization for subqueries. Query plans are optimized by modifying subqueries using semantic knowledge about data. The results of the approach demonstrate the savings in execution cost and the algorithm is more flexible and general compared to existing semantic query optimization methods.

A novel index structure, called All Possible Join tree (APJ-tree) [5], to reduce the searching space for the optimal execution plan of a set of Map Reduce jobs was proposed in the literature. A cost model based RDF join processing solution using Map Reduce to minimize the query responding time was proposed. To speed up join processing a hybrid join and bloom filter are employed. The effectiveness of the cost model was proved in the experimental results performed.

Today cloud computing technology can be used together with semantic web technology to solve many problems. Using this perception, an algorithm to generate the best possible query plan based on a cost model [6] was proposed in the literature. An exhaustive approach to search for best query plan was presented which dynamically determines the number of jobs needed to complete a plan. The results show that the framework is efficient and scalable to handle large number of RDF triples.

A block-oriented dynamic query plan generation approach using pipelined execution was designed. The method consists of two phases, in which a near-optimal plan is chosen by identifying the processing [7] blocks of queries. The second phase uses pipelining concept to generate the optimal plan. Optimization techniques, such as lightweight and fine-grained sideways information passing, semijoin, and other join processing optimizations, were incorporated to further enhance the performance of the query processing engine. Experiments were performed on three different datasets with over billion triples and the results show that the approach outperforms existing RDF query engines.

Computing optimal distributed query plans is a complex problem since the number of possible query plans increases exponentially with respect to the number of relations accessed by the query and due to the increase in the number of sites in a distributed database. To handle this problem, a new algorithm based on multiobjective genetic algorithm [8] was proposed in the literature. The aim of this algorithm is to minimize the total processing cost of query processing. Experiments were performed and the observations show that the algorithm gives better performance and converges quickly towards optimal solutions.

The problem of query optimization [9] and the adaptation of genetic algorithm to the problem of query optimization were described. Four classes of genetic algorithms, that is, basic GA, constraint GA, and heuristic GA, and genetic local search algorithms were implemented and their performances were compared. Binary trees were encoded as chromosomes performing a lot of crossovers. The simulation results proved that the genetic algorithm approach works better compared to traditional query optimization algorithms.

An approach to determine optimal query path [10] based on genetic algorithm was proposed in the literature. Query plans are represented using bushy trees and the trees are encoded using ordinal number encoding. The performance of this algorithm was compared with two-phase optimization and it was proved that genetic algorithm outperforms the two-phase optimization in terms of solution quality, execution time needed, and consistency in performance.

The problem of database query optimization [11] and the adaptation of genetic algorithm to solve the problem were studied in the literature. The algorithm uses a novel encoding scheme by using binary trees to perform crossover operation. The important characteristic of the algorithm is its efficient parallelization. The performance of the proposed algorithm was compared with other algorithms and the test results were documented.

To solve the problem of multijoin query ordering problem [12] which is a part of the query optimizer, an approach which combines the features of Cuckoo and Tabu Search was proposed. The experimental results prove the performance of the proposed algorithm in terms of execution time.

A novel metaheuristics approach [13] was proposed to find the global optimum of continuous global optimization problems with box constraints. The characteristics of modern metaheuristics such as scatter search (SS), genetic algorithms (GAs), and Tabu search (TS) were used in the algorithm. Experiments proved that the approach was quite effective in identifying the global optimum solution to solve the general nonlinear optimization problem.

A new algorithm for solving the problem of multijoin query optimization [14] was designed based on ant colony optimization. The algorithm interprets defining heuristic information, implementing local and global pheromone update, and designing state transition rule. A reasonable solution is obtained after a repeated number of iterations. The simulation results indicate that ant colony optimization was more effective and efficient compared to genetic algorithm.

To optimize RDF chain queries a new algorithm called RDF chain queries using a genetic algorithm (RCQ-GA) [15] was devised in the literature to determine the order in which joins are performed efficiently. A fitness function is defined assuming that the population contains *n* solutions. Ordinal number encoding is used for processing the bushy trees. Experimental results show that the algorithm outperforms other existing approaches in terms of solution quality.

To generate an optimal query plan for the problem of multijoin ordering query optimization problem, a SPARQL Basic Graph Pattern (BGP) query optimization method [16] based on genetic algorithm was designed. The method searches for the optimal query plan in a bushy tree space. The results show that the method can lead to a comparable query performance with considerable optimization time.

## 3. Cuckoo Search Algorithm

Nature inspired metaheuristic algorithms are successfully applied to find solutions for optimization problems. They are characterized by algorithmic operators imitating computationally useful aspects of various natural events. Cuckoo search (CS), Bat algorithm, and Firefly algorithm are examples of some of the nature inspired metaheuristic algorithms.

Cuckoo search (CS) is an optimization technique developed [17] based on the brood parasitism of Cuckoo species by laying their eggs in the nests of other host birds. If a host bird discovers the eggs which are not their own, it will either throw these foreign eggs away or simply abandon its nest and build a new nest elsewhere. This behavior is used in the Cuckoo search algorithm. Each egg in a nest represents a solution, and a Cuckoo egg represents a new solution. The new solution (Cuckoo), if better, is replaced with the solution which is not so good in the nest. In the simplest form of Cuckoo search, each nest contains only one egg. A new solution was generated by Levy flight. The rules for CS are described as follows.

- Each Cuckoo lays one egg at a time and dumps it in a randomly chosen nest.
- The best nests with high quality of eggs will carry over to the next generation.
- The number of available host nests is fixed, and a host can discover a foreign egg with a probability *p*_*a*_ ∈ [0, 1]. In this case, the host bird can either throw the egg away or abandon the nest so as to build a completely new nest in a new location.

The Pseudo code for CS is given in Algorithm 1.

While generating new solution *x*(*t* + 1) for a Cuckoo *i*, a Levy flight is performed using the following:

(1) $$\begin{array}{c} x_{i}^{}\left(t+1\right)=x_{i}^{}\left(t\right)+\alpha ⊕\text{Levy}\left(\lambda \right). \end{array}$$

The symbol ⊕ is an entry-wise multiplication. Basically Levy flights provide a random walk while their random steps are drawn from a Levy distribution for large steps as follows:

(2) $$\begin{array}{c} \text{Levy}\sim u=t^{-\lambda } \end{array}$$

which has an infinite variance with an infinite mean. Here the consecutive jumps of a Cuckoo essentially form a random walk process which obeys a power-law step-length distribution with a heavy tail.

## 4. Proposed Approach

### 4.1. RDF Query Paths

The RDF representation of semantic web data can be queried using SPARQL protocol and RDF query language (SPARQL). Each SPARQL query can be visualized using a query tree. The leaf nodes of a query tree represent any of the triples and the internal nodes are used to join the triples. There are different kinds of representation of query trees like bushy trees, left-deep trees, and right-deep trees. The nodes of these query trees can be structured in many different ways to produce the same results.

**Figure 1:**
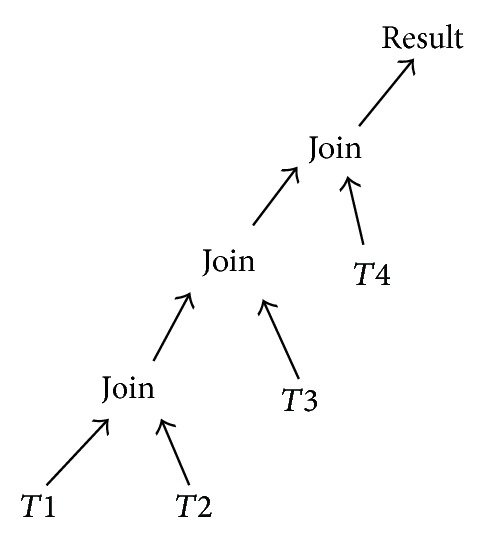
Left-deep trees.

The order in which operations are executed to retrieve the requested data is referred to as query plan or query path. In this research left-deep trees [1] are used. A left-deep tree is a class of join tree with join operators as inner nodes and relations as leaf nodes. For example, Figure 1 shows a left-deep tree which joins *T*1, *T*2, *T*3, and *T*4.

### 4.2. Solution Space

In solution space, each solution represents a query execution plan. The size of the solution depends on the type of tree we use for representing the query plan. Since we use left-deep query tree [1], there are *n*! possible ways to allocate *n* triples to the trees leaves. The leaves of the tree consist of triples and the inner nodes consist of joining of these triples. The *n*! solutions can be obtained by applying the transformation rules like join commutativity, join associativity, left join exchange, and right join exchange.

### 4.3. Encoding

Before any optimization algorithm is applied to solve a problem, a suitable encoding for the solution and a fitness function must be chosen. For query optimization, the solutions are query plans represented using left-deep trees. For encoding left-deep trees, we choose ordered list. Solutions are represented as an ordered list [1] of leaves. For example, for the query plan tree (((*T*1 *∞* *T*2) *∞* *T*3) *∞* *T*4) is encoded as “1234”.

### 4.4. Fitness Function

To determine RDF query path, let us decide on the fitness function. In this research, the fitness function refers to the cost of the left-deep tree. The cost of a left-deep tree depends on the selectivity and cardinality estimation. Consider *R*_*i*_ to be the cardinality and *f*_*i*,*j*_ to be the selectivity. If *p*_*i*,*j*_ is the join predicate between *R*_*i*_ and *R*_*j*_, we can define

(3) $$\begin{array}{c} f_{i,j}=\frac{\left|R_{i}\;\infty_{pi,j}R_{j}\right|}{\left|R_{i}\times R_{j}\right|}. \end{array}$$

For a given join tree *T*, the resultant cardinality |*T*| can be recursively computed as

(4) $$\begin{array}{c} \left|T\right|=\;\left|R_{i}\right|\;\text{if}\;T\;\text{is}\;\text{a}\;\text{leaf}\;R_{i}\\ \left|T\right|=\left(\Pi_{R_{i}\in T1,R_{j}\in T2},f_{i,j}\right)\left|T1\right|\left|T2\right|\;\text{if}\;\;T=T1\;\infty \;T2. \end{array}$$

For a given join tree *T*, the cost function *C*_out_ is defined as

(5) Cout(T)=0 if  T  is  a  leaf RiCout(T)=|T|+Cout(T1)+Cout(T2),if  T=T1  ∞  T2.

## 5. Implementation 

### 5.1. Adaptive Cuckoo Search (ACS) Algorithm for Determining Optimal Query Path

Algorithm 2 is the steps of adaptive Cuckoo search algorithm to solve the problem of query optimization.

To fine tune the convergence rate of Cuckoo search algorithm, parameters like *p*_*a*_, *λ*, and *α* can be modified. The traditional Cuckoo search algorithm uses fixed value for *p*_*a*_, and it cannot be changed dynamically. For a fixed value of *p*_*a*_, it is not possible to find best solutions. The main difference between original Cuckoo search and the adaptive Cuckoo search algorithm is the way in which we modify the value of *p*_*a*_. The value of *p*_*a*_ should be decreased for solutions having fitness less than a threshold and increased for solutions having fitness greater than a threshold. The threshold value is found out by sorting the initial solutions based on their fitness value and finding the median of fitness.

## 6. Dataset Description

Our evaluation is based on three different datasets, namely, Leigh University benchmark (LUBM) dataset, Friend of a Friend (FOAF) dataset, and the Central Intelligence Agency (CIA) World Factbook dataset.

The LUBM benchmark is intended to evaluate the performance of those repositories with respect to extensional queries over a large data set that commits to a single realistic ontology. It consists of university domain ontology, customizable and repeatable synthetic data, a set of test queries, and several performance metrics. The dataset we use here is LUBM5 which consists of about 645,649 triples.

The FOAF consists of millions of RDF documents which describe attributes of people and relationships among them. It consists of about 201,612 RDF triples. The CIA World Factbook contains data about 250 countries defined using RDF statements. Information about government, people, transportation, and many more are provided by this dataset. It consists of more than 100,000 RDF statements.

### 6.1. Experimental Results

Experiments are conducted in a Microsoft Windows XP environment on an Intel Pentium 4 machine with 2GB RAM. Tests are conducted on considering 100,000 triples from each of the three different datasets LUBM, FOAF, and CIA World Factbook. To determine the performance of the algorithm, several experiments are conducted with queries having varying number of predicates. To increase the accuracy of the results, the optimization algorithm is iterated 100 times. The ACS algorithm is compared with the existing algorithms like genetic algorithm (GA), particle swarm optimization (PSO), CS with simple random walk, and CS with Levy flight. The parameters and their values used in the proposed algorithm are given in Table 1.

**Figure 2:**
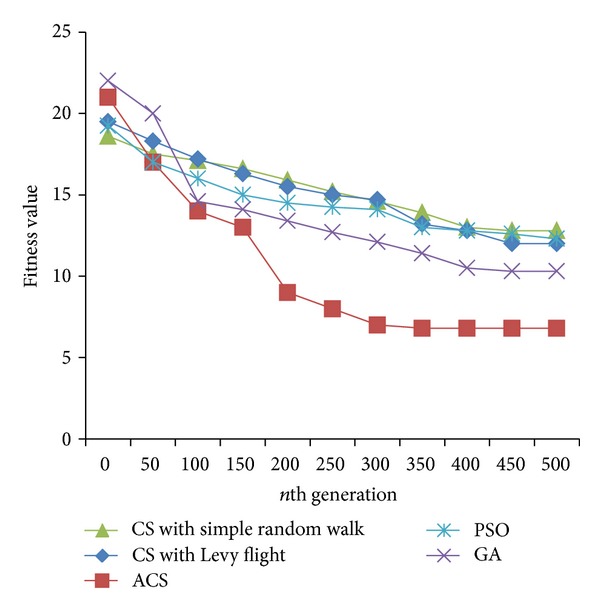
Evolution of best fitness for LUBM dataset.

**Figure 3:**
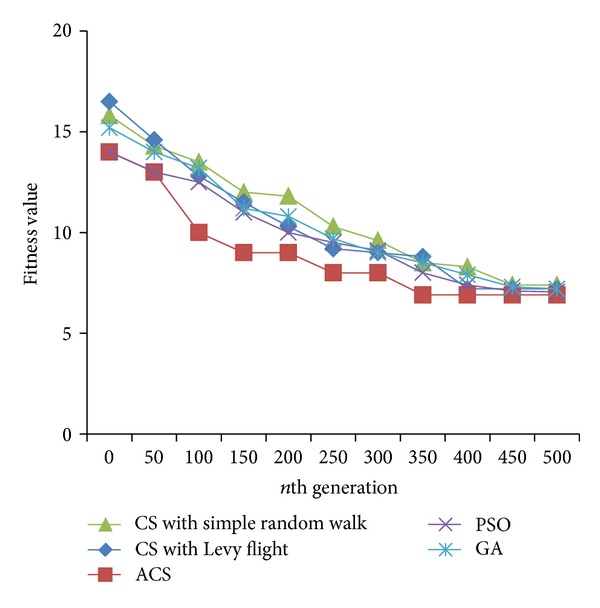
Evolution of best fitness for FOAF dataset.

**Figure 4:**
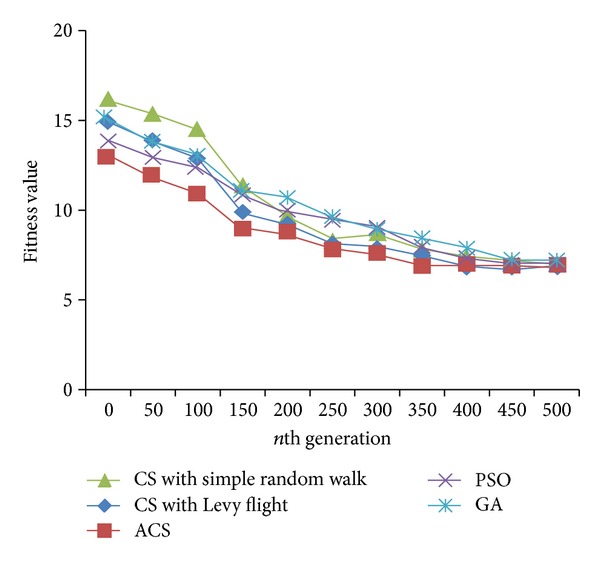
Evolution of best fitness for CIA World Factbook dataset.

Figures 2, 3, and 4 are the fitness charts obtained for the above parameter settings for three different datasets.

The proposed method takes the query cost as fitness function and it is evaluated for 50 to 500 generations to get the optimum cost. From the observations the query cost is decreased gradually when the number of generations increased. Adaptive Cuckoo search algorithm gives optimum results compared with other existing optimization algorithms as shown in Figures 2, 3, and 4. The average execution time is recorded for varying number of predicates and the results are shown.

**Figure 5:**
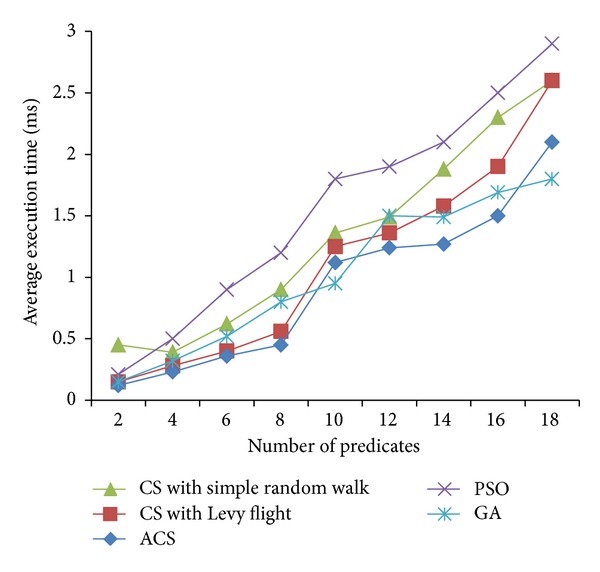
Average execution times (LUBM dataset).

**Figure 6:**
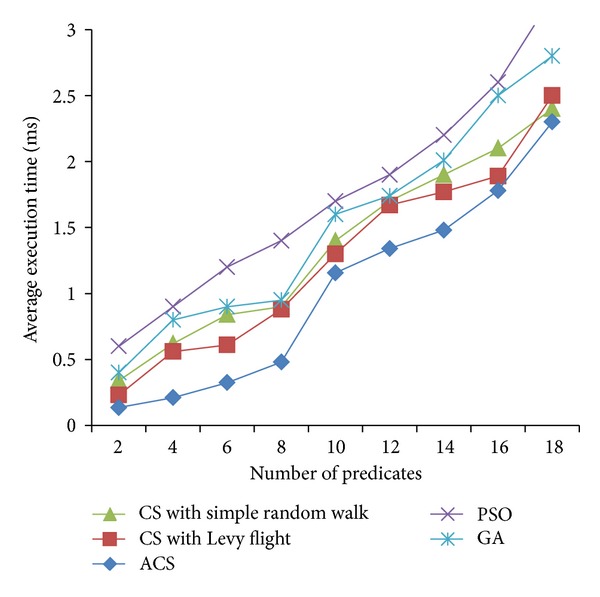
Average execution times (FOAF dataset).

**Figure 7:**
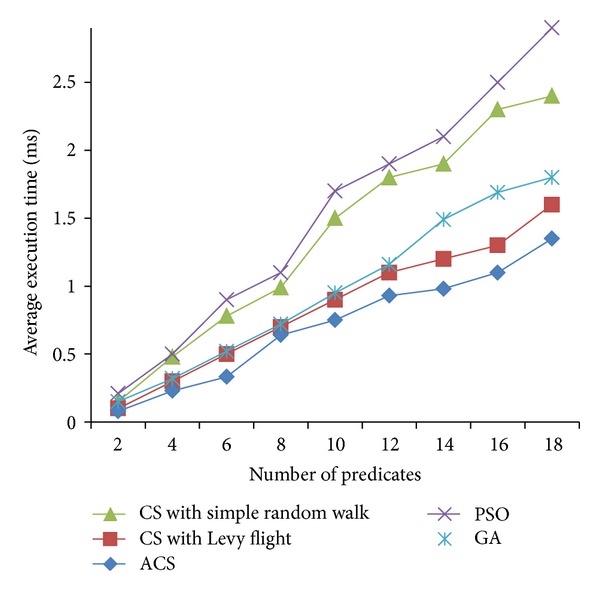
Average execution times (CIA World FactBook dataset).

Figures 5, 6, and 7 show the average execution times of the proposed method compared with PSO, GA, CS with simple random walk, and CS with Levy flight. Execution time linearly increases when the number of predicates is increased in all the five methods. ACS method gives better execution time when compared with other methods because of its less complexity and simple method to find the optimum solution. In particle swarm optimization, the personal best and global best particles are maintained and updated in every iteration and also fitness values are calculated for all new particles generated. So it takes more time to execute to find the optimum results. Genetic algorithm has also several operators like selection, crossover, and mutation which increase the execution time. In adaptive Cuckoo search, number of nests taken is varying from 50 to 500 and the probability of abandoned nest is chosen randomly. Because of less number of populations and the best breeding behavior of Cuckoo species, the proposed algorithm gives optimum results in minimum time compared with other algorithms.

## 7. Conclusions 

In this work, we have presented a novel adaptive Cuckoo search algorithm for the problem of query optimization. The algorithm starts with a solution space consisting of all possible query plans. The fitness function for the algorithm is the cardinality of the triples occurring in the dataset based on the cost model. The cost of the different query plans depends on the order of joins.

The experimental results prove the efficiency of the algorithm. The algorithm has been applied and tested with datasets of varying sizes and the best query plan is generated based on the fitness function. The query execution time is also recorded for diverse dataset sizes. To improve the accuracy of the work, Cuckoo search algorithm can be hybrid with other swarm algorithms in the future and performance can be improved.

## Figures and Tables

**Algorithm 1:**
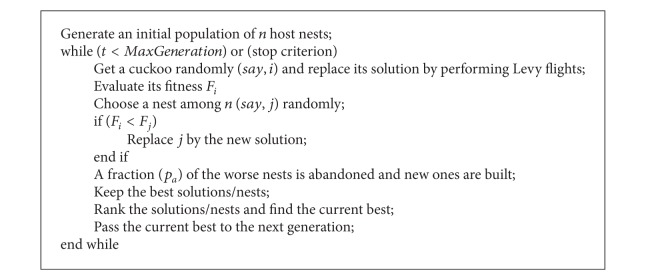
Pseudocode for Cuckoo search.

**Algorithm 2:**
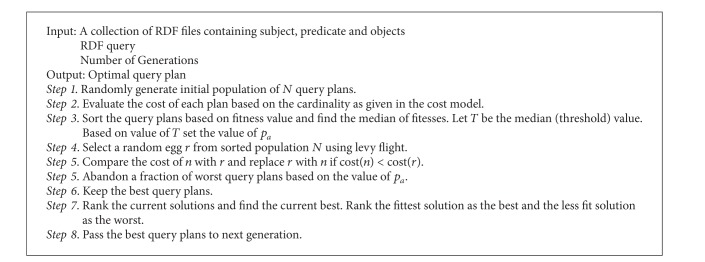


**Table 1:** Parameters and their values for adaptive Cuckoo search algorithm.

Parameter	Value
Adaptive Cuckoo search (ACS)	
Number of nests	50–500
Number of iterations	100
*p*_*a*_	0-1
*α*	1
*λ*	1.5
Number of predicates	2–20

## References

[1] Steinbrunn M, Moerkotte G, Kemper A (1997). Heuristic and randomized optimization for the join ordering problem. *The VLDB Journal*.

[2] Schmidt M, Meier M, Lausen G Foundations of SPARQL query optimization.

[3] Liu C, Wang H, Yu Y, Xu L (2010). Towards efficient SPARQL query processing on RDF data. *Tsinghua Science and Technology*.

[4] Hsu C-N, Knoblock CA (2000). Semantic query optimization for query plans of heterogeneous multidatabase systems. *IEEE Transactions on Knowledge and Data Engineering*.

[5] Zhang X, Chen L, Wang M (2012). Towards efficient join processing over large RDF graph using mapreduce. *Scientific and Statistical Database Management*.

[6] Husain MF, Khany L, Kantarciogluz M, Thuraisingham B Data intensive query processing for large RDF graphs using Cloud Computing tools.

[7] Yuan P, Xie C, Jin H, Liu L, Yang G, Shi X (2014). Dynamic and fast processing of queries on large-scale RDF data. *Knowledge and Information Systems*.

[8] Shina Panicker TV, Kumar V (2014). Distributed query plan generation using multiobjective genetic algorithm. *The Scientific World Journal*.

[9] Dong H, Liang Y Genetic algorithms for large join query optimization.

[10] Hogenboom A, Milea V, Frasincar F, Kaymak U Genetic algorithms for RDF query path optimization.

[11] Horng JT, Kao CY, Liu BJ A genetic algorithm for database query optimization.

[12] Joshi M, Srivastava PR (2013). Query optimization: an intelligent hybrid approach using Cuckoo and Tabu search. *International Journal of Intelligent Information Technologies*.

[13] Trafalis TB, Kasap S (2002). A novel metaheuristics approach for continuous global optimization. *Journal of Global Optimization*.

[14] Li N, Liu Y, Dong Y, Gu J Application of ant colony optimization algorithm to multi-join query optimization.

[15] Hogenboom A, Milea V, Frasincar F, Kaymak U (2009). RCQ-GA: RDF chain query optimization using genetic algorithms. *E-Commerce and Web Technologies*.

[16] Ouyang D, Wang X, Ye Y, Cui X (2012). A GA-based SPARQL BGP reordering optimization method. *Advances in Information Sciences and Service Sciences*.

[17] Yang X, Deb S Cuckoo search via Levy flights.

